# Gas-Assisted Steam Explosion Enables Targeted Regulation of Nutritional and Flavor Quality in *Pleurotus eryngii* via Microstructural Remodeling and Metabolite Modulation

**DOI:** 10.3390/foods15122126

**Published:** 2026-06-12

**Authors:** Dandan Fu, Li He, Yingqi Hu, Jinping Li, Yuyun Lu, Jianzhao Qi, Xinlong Mao, Yanli Huo, Xiangxin Li, Jiayu Dong

**Affiliations:** 1 School of Biological and Pharmaceutical Engineering, Lanzhou Jiaotong University, Lanzhou 730070, China; fdd02272140@163.com (D.F.); 15034184916@163.com (Y.H.); 18626095547@163.com (X.M.); huoylsw@126.com (Y.H.); lixx@mail.lzjtu.cn (X.L.); 17793315951@163.com (J.D.); 2 School of Energy and Power Engineering, Lanzhou University of Technology, Lanzhou 730050, China; lijinping77@163.com; 3 Department of Food Science and Technology, National University of Singapore, Science Drive 2, Singapore 117542, Singapore; fstluy@nus.edu.sg; 4 Center of Edible Fungi, Northwest A&F University, Yangling, Xianyang 712100, China; qjz@nwafu.edu.cn

**Keywords:** Gas-assisted steam explosion, *Pleurotus eryngii*, nutrients, flavor characteristics

## Abstract

Gas-assisted steam explosion (GASE) disrupts raw material structures and promotes active release, but its effects on the nutritional quality and flavor of edible fungi remain unclear. Therefore, this study assessed the influence of GASE on the nutritional quality and flavor characteristics of *Pleurotus eryngii*. Using the sample as the raw material, we selected the GASE process parameters through single-factor experiments combined with response surface methodology and confirmation experiments. Subsequently, changes in nutrient contents and volatile/non-volatile flavor profiles were quantitatively characterized under these processing conditions. The results indicated that the selected parameters effectively disrupted the cell wall structure of the sample, resulting in a loose and porous microstructure. Consequently, the levels of protein, polysaccharides, amino acids and vitamins were significantly altered. In terms of flavor, this process modified the relative odor activity values of key aroma compounds, including volatile aldehydes and pyrazines, while also affecting the distribution of non-volatile metabolites. This led to the enrichment of flavor compounds such as nucleotides and their derivatives, and organic acids. This study confirmed that GASE technology can effectively enhance the nutritional quality and flavor characteristics of the mushroom by regulating its microstructure and metabolite composition.

## 1. Introduction

*Pleurotus eryngii* (commonly named king oyster mushroom or scallop mushroom) is a Basidiomycete fungus belonging to the order Agaricales, family Pleurotaceae [1]. This species is native to southern Europe, especially Italy and Spain [2]. As a highly valued edible fungus, *P. eryngii* is rich in protein, dietary fiber, polysaccharides, and various bioactive compounds [3], which contributes to its widespread popularity in the global health food market [4]. The unique aroma of *P. eryngii* is determined by both volatile and non-volatile compounds, and the types and contents of these components are important indicators for evaluating its quality.

Currently, low-temperature frying and freeze-drying are two prevalent techniques in the processing of edible fungi; however, both methods present significant challenges. Low-temperature frying results in relatively high oil content, which limits its health benefits [5]. In contrast, freeze-drying prevents oil contamination and maintains high product purity, but it suffers from high cost and a dry taste [6], which limit its large-scale production. In this context, steam explosion (SE), a high-temperature, high-pressure instantaneous pressure release, offers a promising alternative for edible fungus processing [7]. SE effectively disrupts the cell wall structure of materials, facilitating nutrient release, improving bioavailability, and enhancing flavor and texture [8].

Existing studies indicate that the effects of SE on the nutritional components of food materials vary depending on the processing targets and parameters. For example, in medicinal plants, while boosting polysaccharide yield from Achyranthes bidentata root, this technique lowers protein content [9], which may be related to protein denaturation and degradation under high temperature and pressure. In crop processing, SE increases the total dietary fiber content and improves its functional properties, such as swelling force and water-holding capacity [10,11,12]. This is attributed to cell wall disruption and the subsequent reconstruction of fiber microstructure [13]. In addition to nutrients, flavor is a core indicator for evaluating food quality and processing palatability. The aroma of edible fungi mainly originates from aldehydes and esters, as well as ketones, alcohols, and heterocyclic compounds [14]. Studies have shown that steam explosion significantly alters the composition and content of flavor compounds in edible fungi. For instance, after steam explosion treatment, shiitake mushrooms show increased levels of aldehydes (imparting nutty and roasted notes), enhancing their characteristic flavor, while some esters decrease due to hydrolysis under high temperature and pressure [15,16,17]. In enoki mushrooms, the SE treatment increases umami amino acids and changes volatile profiles: esters and aldehydes decrease, whereas ketones and pyrazines increase [18].

This study explored the influence of GASE on the nutritional quality (protein, polysaccharides, dietary fiber, etc.) and flavor characteristics (volatile and non-volatile compounds) of *P. eryngii*. By optimizing the process parameters, we revealed the intrinsic relationships among microstructural changes, nutrient transformation, and flavor compound generation, thereby clarifying the effect of GASE on the nutritional and flavor properties of *P. eryngii*. The findings provide a theoretical basis for innovative edible fungus-processing technology, lay a foundation for developing deep-processed *P. eryngii* products with both nutritional and flavor advantages, and support the high-quality development of the edible fungus industry.

## 2. Materials and Methods

### 2.1. Materials and Reagents

*P. eryngii* was obtained from the local farmers’ market in Lanzhou City, Gansu Province. Fresh fruiting bodies were selected based on criteria including intact caps, thick stems, and the absence of mold or rot. Neutral protease, amylase, and cellulase were obtained from Beijing Solarbio Technology Co, Ltd. (Beijing, China). All other chemical and organic reagents were of analytical grade (purity > 99.8%), and the standards were of chromatographic or analytical grade. All reagents met the requirements for analytical laboratory reagents as specified in the national standard GB/T 603-2023 [19].

### 2.2. Gas-Assisted Steam Explosion Treatment

*P. eryngii* of uniform size were selected, thoroughly washed with water, and sliced. The slices were dried in an oven at 45–55 °C until the moisture content reached 10 ± 2%. Subsequently, the dried slices were cut into 1–2 cm^3^ cubes, and 10–15 g of the cubes was accurately weighed and loaded into a GASE apparatus (Lanzhou Jiaotong University). The apparatus was equipped with an independent compressed air pressurization system for GASE. After sealing, compressed air was introduced to reach 0.25 MPa (gauge pressure); then, the chamber was heated to 90 °C, raising the total gauge pressure to 0.7 MPa (water vapor partial pressure about 0.07 MPa, the rest from thermal expansion of air). After holding for 7 min, the pressure was released within 50 ms by rapidly opening a ball valve. All pressures were gauge. The samples were subjected to GASE treatment under various combinations of temperature, pressure, and holding time. After treatment, the samples were dried in an oven at 60 °C to constant weight, and this dry weight was used as the basis for calculating all nutritional yields and contents. The porosity and maximum pore diameter of the samples were observed and measured using scanning electron microscopy (Zeiss, Oberkochen, Germany). Based on single-factor experiments and response surface methodology (RSM), the GASE parameters for *P. eryngii* were selected. The mushroom samples treated under these conditions were then dried, crushed, and passed through an 80-mesh standard sieve, and the resulting powder was reserved for subsequent nutrient extraction experiments.

### 2.3. Nutrient Extraction

#### 2.3.1. Extraction and Content Determination of Crude Polysaccharides

With slight modifications to the method described by Moya et al. [20], 10 g of powder was mixed with distilled water at a solid-to-liquid ratio of 1:30 (g/mL). After adding 0.5% neutral protease (Beijing Solarbio Science & Technology Co., Ltd., Beijing, China, 100 U/mg; one unit is defined as the amount of enzyme that liberates 1 μg of tyrosine from casein per minute at pH 7.0 and 37 °C), the mixture was incubated at 45 °C for 60 min, and then 95 °C for 120 min. The extract was centrifuged (4000 r/min, 10 min), and the supernatant was concentrated under reduced pressure. Four volumes of anhydrous ethanol were added and the mixture was kept at 4 °C overnight for alcohol precipitation. The precipitate was collected by centrifugation and lyophilized to obtain crude polysaccharides.

The polysaccharide content was determined using the 3,5-dinitrosalicylic acid (DNS) [21], with glucose as the standard. Glucose standard solutions were prepared at concentrations of 0, 0.2, 0.4, 0.6, 0.8, and 1.0 mg/mL. Each standard solution was reacted with the DNS reagent, and the absorbance was measured at 540 nm using a UV-Vis spectrophotometer (Shanghai INESA Analytical Instrument Co., Ltd., Shanghai, China), with each concentration measured in triplicate. The calibration curve was plotted with glucose concentration (mg/mL) on the x-axis and absorbance on the y-axis, yielding the linear regression equation: y = 0.7339x − 0.0101 (R^2^ = 0.9913). The sample reaction solution was treated under the same color development conditions, its absorbance was measured, and the crude polysaccharide content was calculated using the calibration curve equation.

#### 2.3.2. Extraction and Content Determination of Crude Protein

With slight modifications to the method described by Liu et al. [22], 10 g of powder was mixed with 0.1 mol/L NaOH at a solid-to-liquid ratio of 1:25 (g/mL), stirred at 45–55 °C for 2.5 h, and ultrasonicated for 20–30 min. After centrifugation (4000 r/min, 15 min), the supernatant was adjusted to pH 4.0 with 1 mol/L HCL and allowed to stand for 2 h for precipitation. The precipitate was collected by centrifugation (8000 r/min, 20 min) and lyophilized to obtain crude protein.

The protein content was determined using the Coomassie Brilliant Blue G-250 method [23]. Bovine serum albumin (BSA) standard solutions were prepared at 0, 0.2, 0.4, 0.6, 0.8, and 1.0 mg/mL. Each standard was reacted with G-250 reagent, and absorbance was measured at 595 nm (triplicate). The calibration curve yielded the linear regression equation y = 0.9197x + 0.0605 (R^2^ = 0.9857). Sample solutions were treated similarly, and the crude protein content was calculated from the calibration curve.

#### 2.3.3. Lipid Extraction and Content Determination

With slight modifications to the method described by Tian et al. [24], 40 g of powder was mixed with n-hexane at a solid-to-liquid ratio of 1:15 (g/mL) and ultrasonicated at 250 W for 60 min, with stirring every 15 min. After standing, the liquid phase was centrifuged at 4000 r/min for 8 min, and the supernatant was concentrated to one-third of its volume by rotary evaporation. Anhydrous ethanol was added, and after standing, the upper layer was removed by aspiration. The precipitate was dried under vacuum to obtain the lipids.

For content determination: the vanillin-phosphoric acid method [25] was used with cholesterol as the standard. Cholesterol standard solutions were prepared at 0, 0.2, 0.4, 0.6, 0.8 and 1.0 mg/mL. Each standard was reacted with the color reagent, and absorbance was measured at 525 nm (triplicate). The calibration curve gave y = 0.6481x − 0.0059 (R^2^ = 0.9921). Sample solutions were measured similarly, and lipid content was calculated.

#### 2.3.4. Dietary Fiber Extraction

Soluble dietary fiber (SDF): With slight modifications to the method described by Zhang et al. [26], 10 g of powder was mixed with distilled water at a solid-to-liquid ratio of 1:20 (g/mL). The pH was adjusted to 4.8, and 2% (*w*/*w*) cellulase (Beijing Solarbio Science & Technology Co., Ltd., Beijing, China, 50 U/mg, one unit is defined as the amount of enzyme that releases 1 μmol of reducing sugar from sodium carboxymethyl cellulose per minute at 50 °C and pH 4.8) was added, and the mixture was incubated at 50 °C for 90 min, followed by enzyme inactivation at 80–90 °C for 10 min. After cooling, the pH was adjusted to 6.0, and equal volumes (1:1, *v*/*v*) of 1% (*w*/*w*) α-amylase (Beijing Solarbio Science & Technology Co., Ltd., Beijing, China, 30 U/mg, one unit is defined as the amount of enzyme that liberates 1 µmol of maltose from starch per minute at pH 6.0 and 25 °C) and neutral protease were added. The mixture was incubated at 50 °C for 60 min, followed by a second enzyme inactivation (80–90 °C, 10 min). After centrifugation (8000 r/min, 15 min), the supernatant was concentrated, mixed with four volumes of 95% ethanol, and kept overnight for alcohol precipitation. The precipitate was vacuum-freeze-dried to obtain SDF.

Insoluble dietary fiber (IDF): IDF from the samples was extracted [27]. The powder was mixed with purified water at a ratio of 1:30 (g/mL), 0.3% (*w*/*w*) thermostable α-amylase was added, and the mixture was incubated at 95 °C, pH 6.0 for 60 min. The temperature was lowered to 50 °C, 0.5% (*w*/*w*) neutral protease was added (pH adjusted to 6.5), and incubation continued for 60 min. Then, the temperature was raised to 60 °C, 0.3% (*w*/*w*) amyloglucosidase (Beijing Solarbio, Science & Technology Co., Ltd., Beijing, China, 100 U/mg; one unit is defined as the amount of enzyme that liberates 1 μmol of glucose from soluble starch per minute at pH 4.5 and 40 °C) was added, and the mixture was incubated for another 60 min. After the enzyme was inactivated at 95 °C for 10 min, the mixture was centrifuged (4000 r/min, 10 min), the supernatant was discarded, and the precipitate was dried to obtain IDF.

#### 2.3.5. Vitamin Determination

Using a liquid chromatography–tandem mass spectrometry (LC-MS/MS) method [28], an appropriate amount of the powder was mixed with 45 mL of ultrapure water and ultrasonicated extraction for 30 min, then centrifuged at 4500 r/min for 15 min. The supernatant was collected, filtered, and the filtrate was diluted to 50 mL. The solution was passed through a 0.45 μm microfiltration membrane (Millipore, Burlington, MA, USA) and analyzed by LC-MS/MS (Agilent Technologies, Inc., Santa Clara, CA, USA). The chromatographic conditions were as follows: column, silica-based pentafluorophenyl (PFP) (1.8 μm particle size, 3.0 mm × 150 mm, Agilent Technologies, Inc., Santa Clara, CA, USA); mobile phase A, 10 mmol/L ammonium formate in 2% formic acid aqueous solution; mobile phase B, 0.1% formic acid in methanol; flow rate, 0.4 mL/min; column temperature, 30 °C; injection volume, 10 μL. Mass spectrometric conditions are detailed in Supplementary Table S1. Quantification was performed using external standard calibration curves. The following MRM transitions were monitored: vitamin B_2_ (377→243), B_3_ (123→80), B_6_ (170→152), and vitamin C (175→115, negative mode).

#### 2.3.6. Amino Acid Determination

The amino acid composition was determined using an amino acid analyzer (Hitachi High-Tech, Tokyo, Japan) [29]. An appropriate amount of the powder was hydrolyzed with 6 mol/L HCl at 110 °C for 22 h to obtain free amino acids. The hydrolysate was then separated on an ion-exchange column and derivatized with ninhydrin. Detection was performed at wavelengths of 570 nm and 440 nm. The amino acid content was calculated using the external standard method (peak area) (1) according to the following equation:

(1) $$C_{i}=\frac{C_{s}}{A_{s}}\times A_{i}$$

*C*_i_- content of amino acid _i_ in the sample test solution (nmol/mL);
*A*_i_-peak area of amino acid _i_ in the sample test solution;
*A*_s_-peak area of amino acid _s_ in the amino acid standard working solution;
*C*_s_-content of amino acid _s_ in the amino acid standard working solution (nmol/mL).

### 2.4. Determination of Volatile Aroma Compounds

Volatile compounds were analyzed by Gas Chromatography-Mass spectrometry (GC-MS, Agilent Technologies, USA). A 500 mg sample was accurately weighed into a headspace vial, mixed with 2 mL of saturated NaCl solution and 20 μL of internal standard solution (3-Hexanone-2,2,4,4-d4 in methanol, 10 μg/mL), and shaken at 60 °C for 5 min. Headspace extraction was performed using a 120 μm DVB/CWR/PDMS fiber at 60 °C for 15 min, followed by desorption at 250 °C for 5 min. A DB-5MS capillary column (30 m × 0.25 mm × 0.25 μm, Agilent J&W Scientific, Folsom, CA, USA) was used. The carrier gas was high-purity helium at a constant flow rate of 1.2 mL/min. Detailed temperature program and mass spectrometry conditions are provided in Supplementary Table S2. The relative odor activity value (rOAV) was used to evaluate the contribution of each compound to the overall flavor. rOAV was calculated according to Equations (2) and (3):

(2) $$C_{i}=\frac{V_{s}\times C_{s}}{M}\times \frac{I_{i}}{I_{s}}\times 10^{-3}$$

(3) $$\mathrm{rOAV}=\frac{C_{i}}{T_{i}}$$

where *C*_i_ is the relative content of the compound (μg/g); *V*_s_ is the volume of the internal standard (μL); *C*_s_ is the concentration of the internal standard (μg/mL); is the sample mass (g); *I*_i_ and *I*_s_ are the peak areas of compound i and the internal standard, respectively. Because both *C*_i_ and *T*_i_ share the same units (μg/g); *T*_i_ is the threshold of the compound (μg/g); rOAV is dimensionless.

### 2.5. Determination of Non-Volatile Taste Compounds

The samples were vacuum-freeze-dried for 63 h and ground into a fine powder. A 50 mg aliquot of the powder was mixed with 1200 μL of a pre-chilled (−20 °C) 70% methanol-in-water containing 2-chloro-L-phenylalanine as internal standard (250 μg/mL), and vortexed six times (once every 30 min, 30 s each). After centrifugation at 12,000 rpm for 3 min, the supernatant was filtered through a 0.22 μm membrane and used for UPLC-MS/MS (Agilent Technologies, Inc., USA) analysis. The column was an Agilent SB-C18 (1.8 μm, 2.1 mm × 100 mm). Mobile phase A was 0.1% formic acid in water and mobile phase B was 0.1% formic acid in acetonitrile. Detailed gradient elution, flow rate, column temperature, and MS parameters are provided in Supplementary Table S3.

### 2.6. Statistical Analysis

Phenotypic data were statistically analyzed using SPSS 26.0, and bar charts were generated using Origin 20.0. Metabolites showing a relative standard deviation (RSD) > 30% across quality control (QC) samples were excluded from further analysis, and the data were log_2_ transformed followed by UV scaling. Pearson correlation analysis was performed to assess sample reproducibility and metabolite correlations. Principal component analysis (PCA) was performed using the Metware Cloud platform (http://cloud.metware.cn accessed on 11 December 2025). Based on the cumulative variance and the Scree plot elbow, the first two principal components (PC1 and PC2) were retained, and the suitability of PCA was confirmed by the clear separation of sample groups in the score plot. Orthogonal partial least squares discriminant analysis (OPLS-DA) was carried out with 7-fold cross-validation and 200 permutation tests. Model validity was assessed using R^2^Y and Q^2^ values, and the model was considered valid when Q^2^ > 0.5. Differential metabolites were screened using three criteria: VIP ≥ 1, |log_2_FC| ≥ 1, and *p* < 0.05.

## 3. Results

### 3.1. Analysis of Microstructural Changes in Pleurotus eryngii Under Gas-Assisted Steam Explosion Treatment

**Figure 1:**
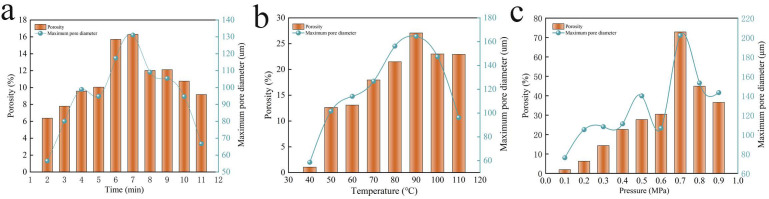
Effect of GASE treatment on the microstructure of *P. eryngii*. (**a**) changes in porosity and maximum pore diameter of *P. eryngii* under different treatment times (2–11 min); (**b**) changes in porosity and maximum pore diameter of *P. eryngii* under different treatment temperatures (40–110 °C); (**c**) changes in porosity and maximum pore diameter of *P. eryngii* under different treatment pressures (0.1–0.9 MPa).

The time, temperature, and pressure of GASE treatment significantly affected the microstructure of *P. eryngii* (Figure 1). With increasing treatment time, both porosity and maximum pore diameter first increased and then decreased, reaching their maximum at 7 min, followed by a gradual decline (Figure 1a). As the temperature increased to 90 °C, porosity and maximum pore diameter increased simultaneously and peaked at this temperature; further temperature increase led to a decrease in both parameters (Figure 1b). Increasing the pressure progressively enhanced porosity and maximum pore diameter, with the maximum achieved at 0.7 MPa; beyond this pressure, both parameters decreased significantly (Figure 1c). Through single-factor experiments and response surface methodology (RSM) screening combined with confirmation experiments (Supplementary Tables S4–S6), the selected GASE conditions were as follows: treatment time of 7 min, temperature of 90 °C, and pressure of 0.7 MPa. Subsequent nutrient extraction and flavor assessment studies were carried out under these conditions.

### 3.2. Analysis of Changes in Nutrient Yield and Content of Pleurotus eryngii Under Gas-Assisted Steam Explosion Treatment

**Figure 2:**
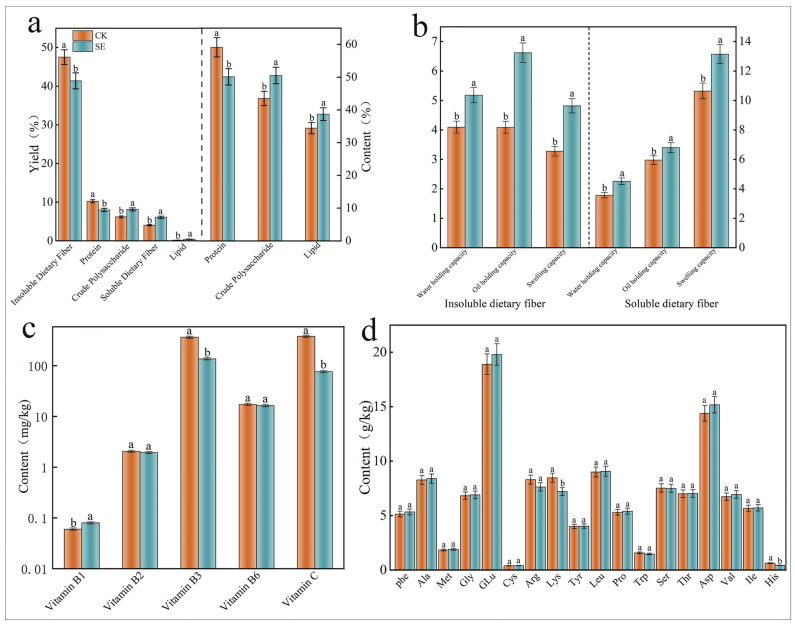
Effects of GASE on nutrient yield and content in *P. eryngii*. (**a**) left panel: changes in the contents of protein, crude polysaccharide, and lipid; right panel: changes in the yields of insoluble dietary fiber, protein, crude polysaccharide, soluble dietary fiber, and lipid under the treatment conditions; (**b**) changes in water-holding capacity, oil-holding capacity, and swelling capacity of insoluble and soluble dietary fibers under the treatment conditions; (**c**) changes in vitamin contents under the treatment conditions; (**d**) changes in amino acid contents under the treatment conditions. Note: Different letters in the figure indicate significant differences between groups (*p* < 0.05).

Under the selected GASE conditions, the nutrient composition of *P. eryngii* exhibited significant changes compared with the control group (CK). Regarding the basic nutritional components, the treatment notably increased the yields of soluble dietary fiber, crude polysaccharides, and fat. Among these, the increases in soluble dietary fiber and crude polysaccharides were particularly pronounced, with the yield of soluble dietary fiber rising by 33.8% and that of crude polysaccharides by 23.7%. Meanwhile, the high-temperature and high-pressure environment induced partial protein degradation, resulting in a 21.7% decrease in protein yield. However, the contents of protein, lipid, and crude polysaccharides increased by 18.02%, 12.37%, and 16.1%, respectively (Figure 2a). In terms of the functional properties of dietary fiber, GASE treatment altered the water-holding capacity, oil-holding capacity, and swelling capacity of both insoluble and soluble dietary fibers (Figure 2b). These improvements are beneficial for enhancing the functional application value of dietary fiber in food processing. Regarding micronutrients, GASE treatment significantly reduced the contents of vitamins B_2_, B_3_, B_6_, and vitamin C. Among these, the most substantial decreases were observed for vitamin B_3_ (a reduction of 227.19 mg/kg) and vitamin C (a reduction of 302.47 mg/kg) (Figure 2c), likely due to the poor thermal stability of these vitamins, leading to their pronounced degradation. On the other hand, the treatment promoted the accumulation of various amino acids, including phenylalanine, alanine, glutamic acid, and aspartic acid. Notably, glutamic acid and aspartic acid increased by 4.76% and 5.56%, respectively (Figure 2d). These two amino acids are key contributors to umami taste. Overall, GASE treatment exerted bidirectional effects on the nutritional quality of *P. eryngii*: soluble dietary fiber, crude polysaccharides, and umami amino acids were increased, whereas protein yield and heat sensitive vitamins were significantly decreased.

Compared with the CK, GASE treatment significantly altered the contents of various carbohydrates in *P. eryngii* (Table 1). Among them, the contents of trehalose, D-sorbitol, D-fructose, and sucrose were significantly increased, while D-mannose, glucose, and D-galactose decreased; no significant differences were observed for D-ribose-5-phosphate, L-rhamnose, D-arabinose, etc. Among all detected carbohydrates, trehalose showed the highest content (328.27 mg/g in CK and 394.80 mg/g in GASE), with an increase of approximately 20.23% after GASE treatment.

### 3.3. Analysis of Changes in Volatile Compounds of Pleurotus eryngii Under Gas-Assisted Steam Explosion Treatment

**Figure 3:**
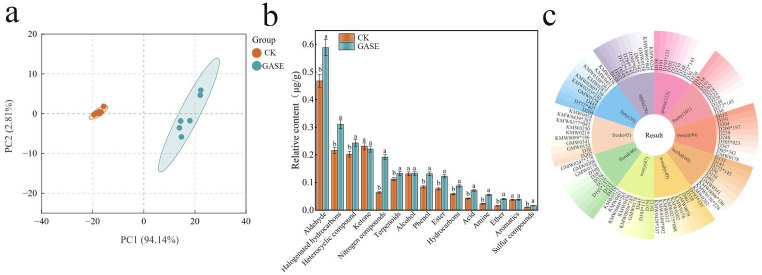
Effects of GASE on volatile metabolites in *P. eryngii*. (**a**) PCA score plot showing the separation between CK and GASE groups; (**b**) Bar chart of relative contents of 15 representative volatile metabolites; (**c**) Flavor wheel of differential metabolites. The central ring indicates the comparison groups (CK vs. GASE). The middle ring lists the top ten sensory descriptors associated with the differential metabolites, with the number of compounds given in parentheses. The outer ring displays the individual differential metabolites. Note: Different letters in the figure indicate significant differences between groups (*p* < 0.05). The asterisks * are used as separators.

Principal component analysis (PCA) showed that the variance contribution rates of PC1 and PC2 were 94.14% and 2.81%, respectively. The sample points of the CK and GASE group formed distinctly separated clusters on the score plot (Figure 3a). Quantitative analysis of flavor substances revealed that the relative contents of aldehydes, halogenated hydrocarbons, and heterocyclic compounds in the GASE group were significantly higher than those in the CK group, with the most prominent increase observed in aldehydes (Figure 3b). Volatile metabolite profiling further indicated that GASE treatment primarily enriched volatile components corresponding to sensory attributes such as fruity (101 compounds), sweet (99 compounds), and herbal (60 compounds), while also retaining a certain proportion of characteristic aroma substances associated with fresh (45 compounds) and floral (46 compounds) notes (Figure 3c).

Based on the analysis of relative odor activity values (rOAV), GASE treatment significantly altered the aroma contribution levels of volatile flavor compounds in *P. eryngii*. (Table 2). Compounds such as 1-nonen-3-one, (E,Z)-2,6-nonadienal, and benzenemethanethiol were identified as core characteristic aroma substances of *P. eryngii*, showing extremely high rOAV values in both the CK and the GASE group. After GASE treatment, the rOAV values of these core compounds further increased: for example, (E,Z)-2,6-nonadienal and benzenemethanethiol increased by 1.7 and 0.7 times. Meanwhile, the rOAV values of most aldehydes, pyrazines, esters, phenols, and sulfur-containing compounds also significantly increased. These changes in rOAV indicate that GASE treatment reshapes the flavor profile of *P. eryngii*, transforming it from a relatively single mushroom-like aroma into a complex aroma characterized by the synergy of green, sweet, roasted, and sulfur notes.

### 3.4. Analysis of Changes in Non-Volatile Taste Compounds of Pleurotus eryngii Under Gas-Assisted Steam Explosion Treatment

**Figure 4:**
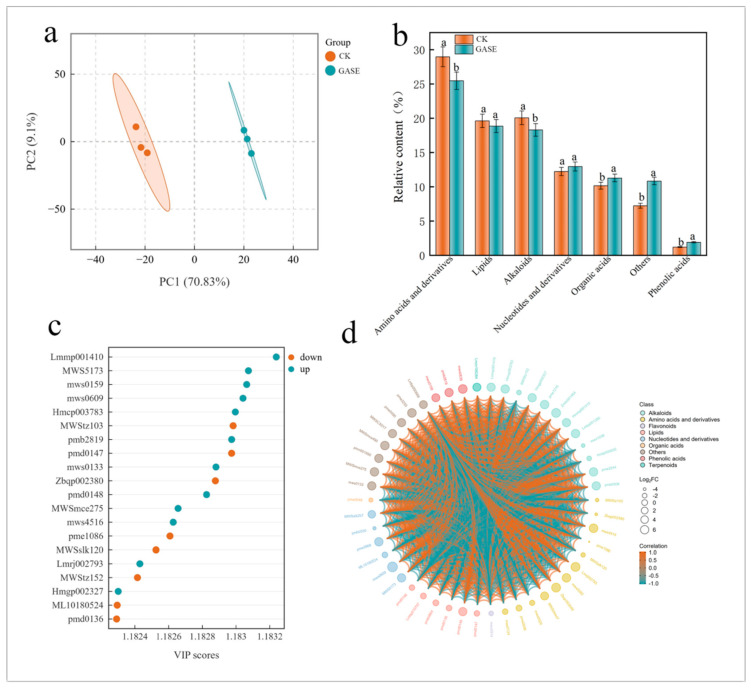
Non-volatile metabolite profiles of *Pleurotus eryngii* under GASE treatment. (**a**) principal component analysis (PCA) score plot; (**b**) relative content expression plot of seven metabolites; (**c**) VIP plot of differential metabolites; (**d**) chord diagram of differential metabolites. Note: Different letters in the figure indicate significant differences between groups (*p* < 0.05).

As shown in Figure 4a, the sample points of the CK and GASE groups formed clearly separated clusters on the PCA score plot, with PC1 and PC2 explaining 70.83% and 9.1% of the total variance, respectively. Classification of metabolites revealed that the relative contents of nucleotides and derivatives, organic acids, other substances, and phenolic acids in the GASE group were significantly higher than those in the CK group, whereas the relative contents of amino acids and derivatives, as well as alkaloids were significantly lower, and no significant difference was observed for lipids between the two groups (Figure 4b). Using an OPLS-DA model validated by 7-fold cross-validation and 200 permutation tests (the original model had R^2^Y = 1.000, Q^2^ = 0.990, all permuted Q^2^ values were lower than the original, *p* < 0.005, confirming no overfitting and good predictive ability; Supplementary Figure S1), variable importance in projection (VIP) analysis identified 22 differential metabolites with VIP > 1.18, of which 14 were up-regulated and 8 down-regulated (Figure 4c). Correlation network analysis further revealed extensive positive and negative correlations among the differential metabolites: amino acids and derivatives together with alkaloids were predominantly negatively correlated, while nucleotides and derivatives together with organic acids were mostly positively correlated (Figure 4d).

### 3.5. Analysis of Changes in Nutrients and Flavor During Gas-Assisted Steam Explosion Treatment

**Figure 5:**
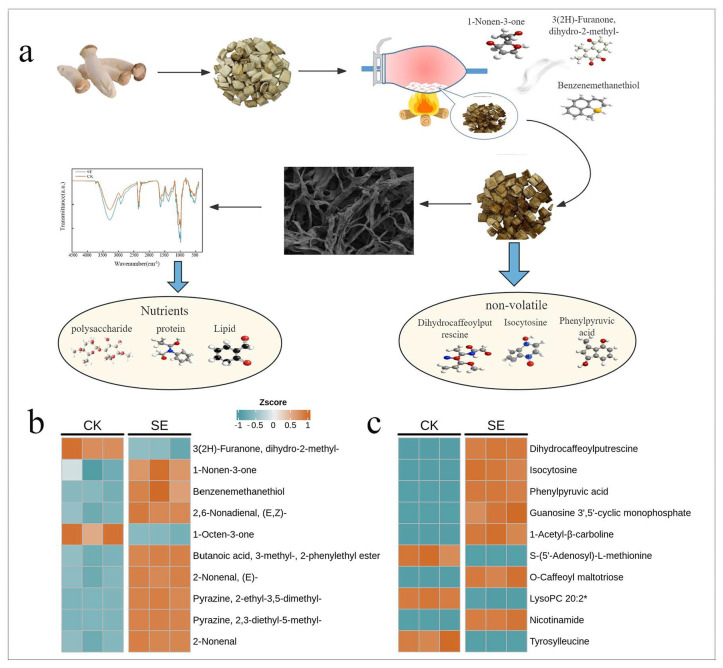
GASE process and metabolite changes. (**a**) Schematic diagram of the GASE treatment process and changes in physicochemical properties of *P. eryngii*; (**b**) Heatmap of changes in volatile flavor compounds; (**c**) Heatmap of changes in non-volatile flavor compounds. Note: * indicates that the structure of the compound is not fully determined (e.g., double-bond positions and geometry are unknown; it may be a mixture of positional isomers). Arrows indicate the direction of the next step in the workflow (step sequence).

GASE treatment reshaped the quality characteristics of *P. eryngii* from multiple dimensions. Regarding microstructure, scanning electron microscopy (Figure 5a) revealed that the fibrous structure of *P. eryngii* became loose and porous after treatment, and Fourier-transform infrared spectroscopy indicated significant changes in the macromolecular functional groups, providing a structural basis for the release and transformation of flavor compounds. Heatmap analysis of volatile flavor compounds (Figure 5b) showed that the normalized abundances of key aroma substances such as benzenemethanethiol, (E,Z)-2,6-nonadienal, and 2-ethyl-3,5-dimethylpyrazine were significantly increased in the GASE group, while the abundance of 1-octen-3-one was decreased. These changes may be related to the enhancement in lipid oxidation, Maillard reaction, and sulfur-containing amino acid degradation during GASE, ultimately leading to the transformation of flavor characteristics such as garlic, cucumber freshness, and roasted nuts. Heatmap analysis of non-volatile substances (Figure 5c) indicated that the abundances of metabolites such as dihydrocaffeoyl putrescine, isocytosine, and phenylpyruvic acid were significantly upregulated in the GASE group, while substances such as S-(5′-adenosyl)-L-methionine and lysophospha-tidylcholine LysoPC 20:2 were significantly downregulated. These changes not only reflect the regulation of the metabolic network of *P. eryngii* by GASE but also provide a material basis for the improvement in its nutritional quality. In summary, GASE drives synergistic changes in volatile and non-volatile metabolites by altering the microstructure of *P. eryngii*, ultimately achieving the observed modifications in flavor and nutritional quality.

## 4. Discussion

Under the selected process parameters (90 °C, 0.7 MPa, 7 min), GASE treatment transformed the dense fibrous structure of *P. eryngii* into a loose and porous morphology, which is consistent with the study by Qiu et al. [31]. The mechanism is as follows: during the high-temperature, high-pressure holding stage, intracellular water becomes superheated; subsequent instantaneous pressure release causes violent water vaporization, and the resulting physical shear forces efficiently disrupt the cell wall structure [32]. This microstructural remodeling effectively breaks the encapsulation of extracellular polymers and significantly increases the contact area for reactants, thereby facilitating the release of internal macromolecular nutrients.

In terms of nutritional quality, the physical tearing of the cell wall restructures the spatial cross-linking pattern of dietary fiber, increasing the yields of insoluble dietary fiber and crude polysaccharides [33,34], and enhancing their functional properties, which is consistent with the study by Han et al. [35]. The accumulation of umami amino acids directly constitutes the taste basis for the enhanced umami flavor of the material [36,37]. The increase in trehalose content after treatment suggests its potential physical-field-induced conversion or retention. Trehalose is a non-reducing sugar generally considered beneficial for maintaining product quality [38], but its actual contribution to long-term storage stability requires further verification. In addition, the intense physical field leads to partial protein degradation and the loss of heat-sensitive vitamins (e.g., vitamin C and B vitamins). This phenomenon is consistent with the findings of Nisov et al. [39,40,41,42]. This indicates that seeking the optimal balance between the release of intracellular components and the protection of heat-sensitive components is the core direction for subsequent technological improvement of this process.

Regarding flavor characteristics, the clear separation between the CK and GASE groups on the PCA score plot indicates that steam explosion reshaped the flavor profile of *P. eryngii*. GASE treatment significantly increased the relative abundance of aldehydes and heterocyclic compounds, which may be attributed to the synergistic effects of lipid degradation and the Maillard reaction during the high-temperature, high-pressure process [43,44]. The rOAV of benzenemethanethiol and (E,Z)-2,6-nonadienal increased markedly, enhancing garlic-like and green notes, respectively. Pyrazine heterocyclic compounds exhibited intense roasted nut and baked aromas, which is consistent with the conclusions of Harada-Padermo et al. [15]. In contrast, the contribution of native aroma compounds such as 1-octen-3-one decreased, possibly due to thermal degradation or conversion into other volatile components at high temperatures, which aligns with the findings of Tu et al. [16].

Among non-volatile taste compounds, the contents of nucleotides and organic acids increased, while the contents of precursor substances such as amino acids and alkaloids decreased correspondingly. The differential metabolite network validated these changes [45,46,47]. This indicates that steam explosion is not merely a mechanical disruption, but rather, through a cascade of “structural remodeling-thermal reaction-metabolic network regulation”, it synergistically achieves targeted regulation of the nutritional and flavor quality of *P. eryngii*.

## 5. Conclusions

This study established the optimal GASE parameters for *P. eryngii*. This process induced microstructural remodeling by disrupting the cell wall, thereby achieving targeted regulation of nutritional and flavor quality. The results showed that GASE significantly increased the yield of insoluble dietary fiber, crude polysaccharides, and the functional properties of dietary fiber, and promoted the enrichment of umami amino acids and trehalose. In terms of flavor, GASE treatment significantly increased the relative odor activity values (rOAV) of aldehydes and pyrazines while also reshaping the non-volatile taste metabolite profile. These changes may be related to the enhancement in the Maillard reaction and lipid oxidation. This reveals for the first time the molecular mechanism of GASE in synergistically optimizing the nutritional and flavor quality of *P. eryngii* through a cascade of “structural disruption-thermal reaction-metabolic network regulation”. Future research should focus on the development of fat-reducing and umami-enhancing seasoning products.

## Figures and Tables

**Table 1:** Carbohydrate contents in *Pleurotus eryngii* under GASE treatment.

No	Substance	CK	GASE
1	Cellobiose	0.014 ± 0.001 ^b^	0.079 ± 0.011 ^a^
2	D-ribose-5-phosphate	0.196 ± 0.047 ^a^	0.089 ± 0.050 ^a^
3	L-Rhamnose	0.005 ± 0.002 ^a^	0.004 ± 0.0002 ^a^
4	D-Mannose	0.141 ± 0.002 ^a^	0.037 ± 0.004 ^b^
5	Levoglucosan	0.326 ± 0.009 ^b^	0.370 ± 0.006 ^a^
6	Inositol	0.598 ± 0.021 ^a^	0.611 ± 0.093 ^a^
7	Glucose	0.497 ± 0.010 ^a^	0.188 ± 0.026 ^b^
8	D-Sorbitol	0.262 ± 0.025 ^b^	0.665 ± 0.139 ^a^
9	D-Galactose	0.008 ± 0.001 ^a^	0.006 ± 0.0003 ^b^
10	2-Acetamido-2-deoxy-D-glucopyranose	0.047 ± 0.005 ^a^	0.049 ± 0.007 ^a^
11	Trehalose	328.267 ± 2.647 ^a^	394.796 ± 65.184 ^b^
12	Sucrose	0.001 ± 0.0003 ^b^	0.004 ± 0.0007 ^a^
13	D-Arabinose	0.003 ± 0.0007 ^a^	0.003 ± 0.0008 ^a^
14	D-Arabinitol	0.049 ± 0.002 ^b^	0.080 ± 0.006 ^a^
15	D-Fructose	0.122 ± 0.002 ^b^	0.174 ± 0.029 ^a^
16	Xylitol	0.027 ± 0.001 ^b^	0.044 ± 0.004 ^a^

Note: Different lowercase letters in the table indicate significant differences (*p* < 0.05).

**Table 2:** Odor contribution of volatile aroma components in *Pleurotus eryngii* under GASE treatment.

No	Compounds	Odor	Threshold	rOAV	
				CK	GASE
1	3(2H)-Furanone, dihydro-2-methyl-	sweet, solvent, bread, buttery, nutty	5 × 10^−^^6^	592,743.511	381,299.380
2	2(5H)-Furanone, 5-ethyl-3-hydroxy-4-methyl-	sweet, fruity, caramel, maple, fenugreek, brown, sugar, nutty, chicory, praline, butterscotch	2 × 10^−6^	160,370.818	271,397.434
3	2,6-Nonadienal, (E,Z)-	cucumber, green	1 × 10^−5^	8751.459	23,452.611
4	1-Nonen-3-one	pungent, mushroom	1 × 10^−6^	12,153.289	16,262.774
5	Benzenemethanethiol	sharp, alliaceous, onion, sulfury, garlic, horseradish, minty, coffee	3.5 × 10^−6^	8254.388	14,160.250
6	2-Nonenal, (E)-	fatty, green, cucumber, citrus	8 × 10^−5^	1988.206	4672.720
7	1-Octen-3-one	mushroom	5 × 10^−6^	6960.456	4382.446
8	2-Hexenal, (E)-	green, grassy	0.0031	2786.706	3801.392
9	2-Nonenal	fatty, green, waxy, cucumber, melon	0.0001	1590.565	3738.176
10	1-Butanol, 3,3-dimethyl-	-	8.2 × 10^−5^	5942.141	3110.827
11	Non-8-enal	smoky, plastic	0.0002	1686.932	2991.587
12	Pyrazine, 2-ethyl-3,5-dimethyl-	burnt, almond, roasted, nutty, coffee	4 × 10^−5^	204.994	1951.431
13	Butanoic acid, 3-methyl-, 2-phenylethyl ester	floral, fruity, sweet, rose, peach, apricot	1 × 10^−5^	362.241	1476.588
14	Pyrazine, 2,3-diethyl-5-methyl-	musty, nut skin, earthy, roasted, hazelnut, toasted, potato, dusty, foliage, vegetable	3.1 × 10^−5^	109.553	1435.447
15	2-Furanmethanethiol, 5-methyl-	sulfury, roasted, coffee	5 × 10^−5^	215.089	1231.971
16	3-Octen-2-one	earthy, spicy, herbal, sweet, mushroom, hay, blueberry	3 × 10^−5^	2433.880	1009.233
17	2-octenal	fatty, green, herbal	0.0002	566.453	864.919
18	2-Heptanone, 6-methyl-	camphor	0.0081	1343.376	690.052
19	2-Hexenal	sweet, almond, fruity, green, leafy, apple, plum, vegetable	0.017	467.448	678.022
20	trans,cis-2,6-Nonadien-1-ol	green, cucumber, oily, violet, leafy	0.001	233.252	656.416
21	Methyl ethyl disulfide	sulfury, truffle	6.2 × 10^−5^	334.334	485.990
22	2,6-Nonadienal, (E,E)-	fresh, citrus, green, cucumber, melon	0.0005	136.336	459.982
23	6-Nonenal, (E)-	-	2.2 × 10^−5^	277.139	441.614
24	(Z,Z)-3,6-Nonadienal	fatty, soapy, cucumber	5 × 10^−5^	692.557	416.630
25	4-(2,6,6-Trimethylcyclohexa-1,3-dienyl)but-3-en-2-one	-	9 × 10^−5^	103.947	404.092
26	Octane, 1-iodo-	-	0.0002	161.505	286.402
27	2-Methylisoborneol	earthy, musty	0.00048	202.211	269.216
28	Phenol, 2-methoxy-	nutty	0.0016	119.357	224.755
29	Hexanal	aldehyde, grassy, green, leafy, vinegar	0.005	350.062	195.670
30	Butanoic acid, 3-methyl-, ethyl ester	strawberry, candy, fruity	0.0001	128.050	185.962
31	2-Hexanone, 4-methyl-	-	0.00081	119.938	169.222
32	Benzaldehyde, 4-methoxy-	sweet, powdery, mimosa, floral, hawthorn, balsamic	0.0002	111.112	148.448
33	p-Cresol	phenol, narcissus, animalic, mimosa	0.00024	13.040	128.165
34	2-Butanethiol, 3-methyl-	repulsive, beefy, meaty	0.0002	124.538	118.128
35	3-Mercaptohexyl acetate	sulfury, grapefruit, fruity	2 × 10^−5^	57.375	108.749
36	Oxazole, trimethyl-	nutty, nut skin, roasted, wasabi, shellfish, mustard, vegetable	0.005	61.964	89.414
37	2-Nonenal, (Z)-	orris, fatty, waxy, cucumber	0.0045	35.346	83.071
38	6-Nonenal, (Z)-	green, cucumber, melon, cantaloupe, honeydew, waxy, vegetable, orris, violet, leafy	0.00014	43.550	69.397
39	Nonanal	aldehyde, citrus, orange peel	0.001	76.623	62.406
40	2-Octenal, (E)-	fresh, cucumber, fatty, green, herbal, banana, waxy, leafy	0.003	37.764	57.661
41	2(5H)-Furanone, 5-ethyl-	spice	0.0097	109.740	55.335
42	Paraldehyde	sweet, aromatic	0.02	51.188	52.178
43	Phenol, 2-methyl-	phenol	0.0039	8.267	49.102
44	Germacrene D	woody, spice	0.0012	11.668	45.968
45	Ethyl maltol	sweet, caramel, jammy, strawberry, cotton, candy	0.044	29.743	43.115
46	3-Nonanone	caramel, spicy, sweet	0.017	4.074	39.614
47	2-Pentanol, acetate	herbal, weedy, musty, green, vegetable, nut skin, beany, ketonic, animalic	0.015	24.917	34.914
48	Pyrazine, 3-ethyl-2,5-dimethyl-	potato, cocoa, roasted, nutty	0.0086	3.460	33.750
49	Geraniol	sweet, floral, fruity, rose, waxy, citrus	0.0066	21.185	31.761
50	Hexanethioic acid, S-methyl ester	cabbage, rubbery	0.0003	20.773	29.657
51	Benzene, n-butyl-	-	0.014	35.373	27.888
52	3-Methylthiobutyraldehyde	cooked potato	0.0025	22.237	25.518
53	2(3H)-Furanone, 5-hexyldihydro-	fresh, oily, waxy, peach, coconut, buttery, sweet	0.0011	5.372	21.301
54	Benzeneacetaldehyde	floral, honey, rose, cherry	0.0063	7.093	20.538
55	Phenol	phenol, medicinal	0.03	19.314	20.126

Note: rOAV values are semi-quantitative estimates based on internal standard calibration and are primarily intended for comparing the relative aroma contribution of compounds within the current dataset. Odor thresholds were mainly obtained from the compendium by [30]; those not available therein were retrieved from the VCF database (https://www.vcf-online.nl accessed on 9 July 2025).

## Data Availability

The original contributions presented in this study are included in this article/Supplementary Materials. Further inquiries can be directed to the corresponding author.

## Supplementary Materials

The following supporting information can be downloaded at: https://www.mdpi.com/article/10.3390/foods15122126/s1, Tables S1–S3: Operating parameters for LC-MS/MS, GC-MS, and UPLC-MS/MS; Tables S4–S6: Response surface optimization results: ANOVA table, regression equation, model fit statistics, coded levels, and the equation in terms of actual factors; Table S7: Volatile metabolomics data; Table S8: Non-volatile metabolomics data. Figure S1: OPLS- DA permutation plot.
