# Supplementary material for: Gas-Assisted Steam Explosion Enables Targeted Regulation of Nutritional and Flavor Quality in Pleurotus eryngii via Microstructural Remodeling and Metabolite Modulation
Source: Foods. 2026 Jun 12;15(12):2126. doi: 10.3390/foods15122126 (PMC13297972; doi:10.3390/foods15122126)
Supplement: Supplementary file 1 [file foods-15-02126-s001.zip › Figure S1.pdf]

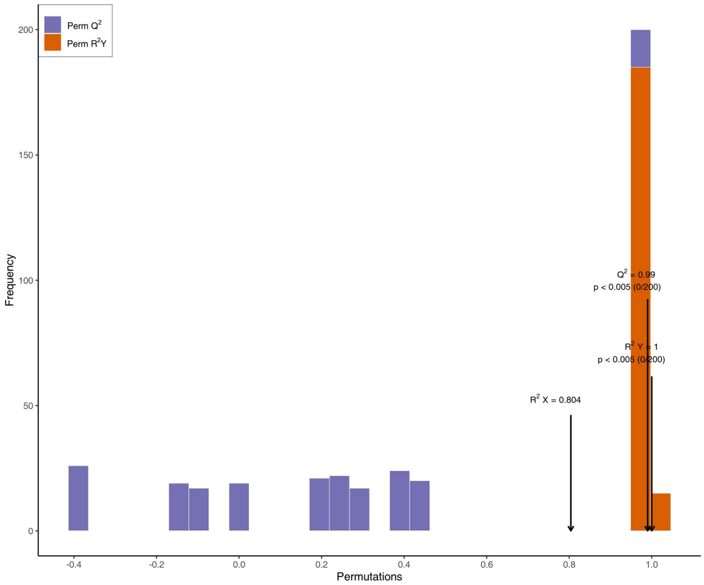

Figure S1. OPLS- DA permutation plot

Note: The x-axis represents the R^2Y and Q^2 values of the model, and the y-axis represents the frequency of model classification effects observed in 200 random permutation experiments. Orange dots represent the R^2Y values of randomly permuted models, purple dots represent the Q^2 values of randomly permuted models, and the black arrows indicate the R^2X, R^2Y, and Q^2 values of the original model.
