# Supplementary material for: Gas-Assisted Steam Explosion Enables Targeted Regulation of Nutritional and Flavor Quality in Pleurotus eryngii via Microstructural Remodeling and Metabolite Modulation
Source: Foods. 2026 Jun 12;15(12):2126. doi: 10.3390/foods15122126 (PMC13297972; doi:10.3390/foods15122126)
Supplement: Supplementary file 1 [file foods-15-02126-s001.zip › Table S1-Table S3.pdf]

Table S1. Mass spectrometric conditions for LC-MS/MS analysis

Parameter	Setting
Instrument	Agilent 6460 triple quadrupole
Ion source	Electrospray ionization (ESI), positive/negative switching
Ion source temperature	500 °C
Spray voltage	5500 V (positive) / –4500 V (negative)
Sheath gas (GS1)	50 psi
Auxiliary gas (GS2)	60 psi
Curtain gas (CUR)	25 psi
Collision energy	35 eV
Acquisition mode	Multiple reaction monitoring (MRM)

Table S2. GC- MS temperature program and MS conditions

Parameter	Setting
Oven temperature program	Hold at 40 °C for 3.5 min; ramp at 10 °C/min to 100 °C; then at 7 °C/min to 180 °C; finally at 25 °C/min to 280 °C, hold for 5 min
Inlet temperature	250 °C
Solvent delay	3.5 min
Ion source	EI, 230 °C
Quadrupole temperature	150 °C
Transfer line temperature	280 °C
Electron energy	70 eV
Scan mode	Selected ion monitoring (SIM)

Table S3. UPLC- MS/MS gradient elution and mass spectrometry parameters

Parameter	Setting
Flow rate	0.35 mL/min
Column temperature	40 °C

Parameter	Setting
Injection volume	2 μL
Gradient program	Time (min) | B phase (%)<br>0.00 | 5<br>9.00 | 95 (linear increase)<br>10.00 | 95 (hold 1 min)<br>11.10 | 5<br>14.00 | 5 (equilibration)
Ion source	ESI
Ion source temperature	500 °C
Ion spray voltage (IS)	5500 V (positive) / -4500 V (negative)
Gas settings	GS1: 50 psi, GS2: 60 psi, CUR: 25 psi
Collision- induced ionization	High
