# Supplementary material for: Gas-Assisted Steam Explosion Enables Targeted Regulation of Nutritional and Flavor Quality in Pleurotus eryngii via Microstructural Remodeling and Metabolite Modulation
Source: Foods. 2026 Jun 12;15(12):2126. doi: 10.3390/foods15122126 (PMC13297972; doi:10.3390/foods15122126)
Supplement: Supplementary file 1 [file foods-15-02126-s001.zip › Table S4-Table S6.pdf]

In this study, a Box- Behnken design with three factors and three levels was adopted, and the coded levels of the independent variables are as follows:

Table S4 Response surface model and coded levels

	Name	Units	Low	High
A	Time	Min	2	11
B	Pressure	MPa	0.1	0.9
C	Temperature	℃	40	110

Table S5 Experimental Design and Results of Response Surface Methodology for Porosity of Pleurotus eryngii

Experimental No	Time（min）	Pressure（MPa）	Temperature（℃）	Porosity （%）
1	2	0.1	60	12.35
2	2	0.5	80	25.67
3	2	0.9	100	32.46
4	4	0.1	80	18.90
5	4	0.5	100	38.23
6	4	0.1	60	42.57
7	6	0.9	100	28.13
8	6	0.1	80	45.79
9	6	0.5	90	52.12
10	7	0.7	90	58.48
11	7	0.9	80	48.90
12	7	0.1	60	32.35
13	7	0.7	90	62.77
14	8	0.5	80	46.68
15	8	0.9	100	45.34
16	10	0.1	100	32.68
17	10	0.5	60	42.13

Table S6. Analysis of Variance (ANOVA)

Source	Sum of Squares	df	Mean Square	F- value	p- value	Significance
Model	2741.12	9	304.57	12.33	0.0016	**
A- Time	88.25	1	88.25	3.57	0.1007	
B- Pressure	545.11	1	545.11	22.06	0.0022	**
C- Temperature	4.51	1	4.51	0.18	0.6819	
AB	106.81	1	106.81	4.32	0.0762	
AC	86.38	1	86.38	3.50	0.1037	
BC	35.08	1	35.08	1.42	0.2723	
A^2	360.78	1	360.78	14.60	0.0065	**
B^2	303.58	1	303.58	12.29	0.0099	**
C^2	3.71	1	3.71	0.15	0.7100	
Residual	172.98	7	24.71			
Lack of Fit	163.59	6	27.27	2.90	0.4213	
Pure Error	9.39	1	9.39			
Cor Total	2914.10	16				

Note: *, $P < 0.05$, significant difference; , $P < 0.01$, highly significant difference.

Regression Equations: Coded factors equation (for comparing factor effects):

$$\text{Porosity (\%)} = 51.62 + 5.55A + 8.88B + 1.77C - 7.06AB + 10.57AC + 4.27BC - 16.99A^2 - 9.04B^2 - 3.87C^2$$

Actual factors equation (for prediction): $\text{Porosity (\%)} = -8.401 + 9.129\times\text{Time} + 94.777\times\text{Pressure} - 0.0689\times\text{Temp} - 4.485\times\text{Time}\times\text{Pressure} + 0.0671\times\text{Time}\times\text{Temp} + 0.3486\times\text{Pressure}\times\text{Temp} - 0.8392\times\text{Time}^2 - 73.774\times\text{Pressure}^2 - 0.003157\times\text{Temp}^2$

Model Diagnostics and Validation:

Lack-of-fit: $F=2.90$, $p=0.4213 > 0.05$, not significant → model is adequate.

Difference between Pred R^2 and Adj R^2: Pred R^2 (0.082) vs Adj R^2 (0.864) suggests possible overfitting, therefore confirmation runs were performed.

Predicted optimum: Time = 7.02 min, Pressure = 0.71 MPa, Temperature = 90.3 °C, Predicted porosity = 60.85%.

Confirmation experiments: Three replicates at the practical conditions (7 min, 0.7 MPa, 90 °C) gave an average porosity of 60.63 ± 2.15%, with a relative error <1% compared to the predicted value, confirming model reliability.

Desirability Function:
In this study, only a single response (porosity) was optimized, and no multi- response optimization was performed; therefore, the desirability function was not calculated. If maximizing porosity is desired, the optimal point can be directly predicted from the regression equation.
